# Pectus Excavatum and Risk of Right Ventricular Failure in Left Ventricular Assist Device Patients

**DOI:** 10.31083/j.rcm2411313

**Published:** 2023-11-09

**Authors:** Casper F. Zijderhand, Yunus C. Yalcin, Jelena Sjatskig, Daniel Bos, Alina A. Constantinescu, Olivier C. Manintveld, Ozcan Birim, Jos A. Bekkers, Ad J. J. C. Bogers, Kadir Caliskan

**Affiliations:** ^1^Department of Cardiothoracic Surgery, Erasmus MC, University Medical Center Rotterdam, 3015 GD Rotterdam, The Netherlands; ^2^Department of Cardiology, Erasmus MC, University Medical Center Rotterdam, 3015 GD Rotterdam, The Netherlands; ^3^Department of Radiology and Nuclear Medicine, Erasmus MC, University Medical Center Rotterdam, 3015 GD Rotterdam, The Netherlands; ^4^Department of Epidemiology, Erasmus MC, University Medical Center Rotterdam, 3015 GD Rotterdam, The Netherlands

**Keywords:** chest-wall abnormalities, pectus excavatum, right ventricle failure, left ventricular assist device, readmission

## Abstract

**Background:**

Right ventricular failure (RVF) is a significant cause of morbidity and mortality in patients with a left ventricular assist device (LVAD). This study is aimed to investigate the influence of a pectus excavatum on early and late outcomes, specifically RVF, following LVAD implantation.

**Methods:**

A retrospective study was performed, that included patients with a HeartMate 3 LVAD at our tertiary referral center. The Haller index (HI) was calculated using computed tomography (CT) scan to evaluate the chest-wall dimensions.

**Results:**

In total, 80 patients (median age 57 years) were included. Two cohorts were identified: 28 patients (35%) with a normal chest wall (HI <2.0) and 52 patients (65%) with pectus excavatum (HI 2.0–3.2), with a mean follow-up time of 28 months. Early (≤30 days) RVF and early acute kidney injury events did not differ between cohorts. Overall survival did not differ between cohorts with a hazard ratio (HR) of 0.47 (95% confidence interval (CI): 0.19–1.19, *p * = 0.113). Late (>30 days) recurrent readmission for RVF occurred more often in patients with pectus excavatum (*p* = 0.008). The onset of late RVF started around 18 months after implantation and increased thereafter in the overall study cohort.

**Conclusions:**

Pectus excavatum is observed frequently in patients with a LVAD implantation. These patients have an increased rate of readmissions and late RVF. Further investigation is required to explore the extent and severity of chest-wall abnormalities on the risk of RVF.

## 1. Introduction

Left ventricular assist devices (LVAD) have become an accepted treatment modality to improve survival, functional capacities, and quality of life in patients with end-stage heart failure [1, 2]. Technological improvements and increasing clinical implantations have led to further improvements in LVAD therapy outcomes [3]. Nevertheless, serious early and late adverse events following LVAD implantation hamper favorable clinical outcomes and lead to significant morbidity and mortality in LVAD-supported patients [4]. Such adverse events include bleeding, infection, right ventricular failure (RVF), device malfunction, cerebrovascular accidents, and renal failure [5]. One of the significant drivers of morbidity and mortality is early onset RVF or progressive decline of the right ventricular function after LVAD implantation [6]. RVF occurs in up to 42% of patients post-LVAD implantation, depending on the diagnostic criteria used [7]. RVF is a harbinger of insufficient LVAD flow, resulting in decreased tissue perfusion, acute renal injury, and multi-organ failure [8].

The right ventricular function may be compromised by mechanical and anatomical compression of the LVAD outflow graft, especially the part with stiff bend relief [9]. Chest-wall abnormalities, such as pectus excavatum, could predispose to RVF by increasing the pressure directly on the right heart and the LVAD and corresponding components [10]. This potentially influences the right ventricular function causing constrictive physiology with elevated central venous pressure and right-sided congestion, resulting in compromised renal function, hepatic dysfunction, and systemic congestion [11, 12, 13].

To prevent the often-devastating consequences of RVF it is essential to identify and understand the underlying mechanisms that can cause RVF after LVAD implantation. To date, only limited data have been published on the impact of pectus excavatum on outcomes following LVAD implantation, including the incidence of RVF. This study therefore aimed to investigate the influence of a pectus excavatum on early and late outcomes, specifically RVF, following LVAD implantation.

## 2. Methods

### 2.1 Study Design and Data Collection

The hospital records were retrospectively reviewed of all adult patients (≥18 years) who received an LVAD between January 2016 and December 2020 in the Erasmus Medical Center. Our HeartMate 3 LVAD program started at the beginning of 2016, and therefore only patients with a HeartMate 3 device were enrolled in this study. Patients were eligible for inclusion if they underwent a pre- or postoperative computed tomography (CT) scan of their chest. Patient characteristics before LVAD implantation, procedural characteristics, and outcomes were collected from the local data input of the European Registry for Patients with Mechanical Circulatory Support (EUROMACS) database. If data was missing from the database, then it would be replaced with local electronic health record data until all data fields were populated. Length of hospital stay included the day of LVAD implantation until the patients’ discharge date.

### 2.2 Computed Tomography Analysis

**Fig. 1.**
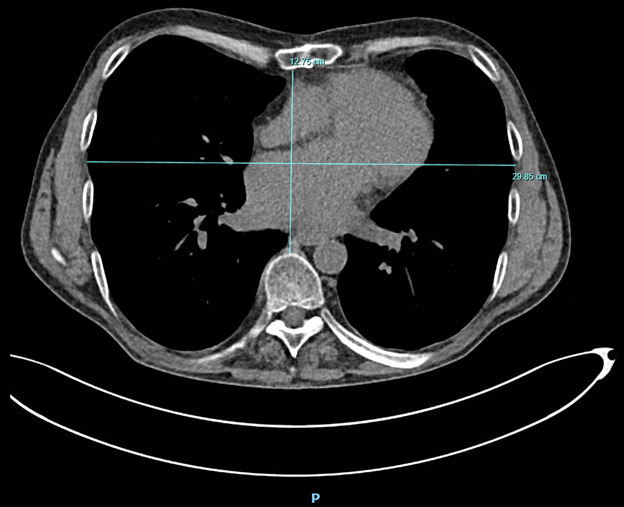
**An axial computed tomography (CT) scan of a 58-year-old male patient, before left ventricular assist device (LVAD) implantation**. The maximal transverse diameter is 29.85 centimeters (cm) and minimum anterior posterior distance is 12.75 cm. Haller index; 29.85/12.75 = 2.34, categorized as Haller index 2, mild pectus excavatum. P, posterior side of the patient.

Patients were stratified, according to their chest CT scan, into 4 groups based on their Haller index (HI) [14]. In the axial plane, the HI was calculated as the maximum transverse diameter of the chest wall divided by the minimum anterior posterior distance between the sternum and vertebrae (Fig. 1). Group 1 was defined as a normal chest with a HI <2.0, group 2 mild excavatum with a HI 2.0–3.2, group 3 moderate excavatum with a HI 3.2–3.5 and group 4 severe excavatum with a HI >3.5 [14].

### 2.3 Outcomes

The primary outcome was early (≤30 days) and late (>30 days) RVF post-LVAD implantation. Early RVF was defined as either receiving short- or long-term right-sided circulatory support or the need for continuous inotropic support for ≥14 days [15]. Late RVF was defined as heart failure requiring readmission and medical or surgical intervention after initial surgery. Secondary outcomes included bleeding events, neurological dysfunction, acute kidney injury (AKI), chronic kidney disease (CKD), overall readmission, readmission for RVF, and overall mortality.

### 2.4 Statistical Analysis

Baseline categorical data were presented as percentages and compared with the Chi-squared test or Fisher’s exact test, in case of a cell frequency of <5. Baseline continuous variables were presented as median with interquartile range (non-Gaussian) and normality was tested using the Shapiro–Wilk test. To identify potential early (≤30 days) adverse events a univariable logistic regression analysis was performed, and factors were presented as odds ratio (OR). A *p* value of < 0.05 was considered statistically significant. Late (>30 days) RVF and overall survival, stratified by pectus excavatum, were calculated and presented as Kaplan–Meier plots. Differences were compared by a log-rank test. Time-to-event analysis for late outcomes was performed with a univariable Cox regression and data was presented as hazard ratio (HR). In addition, a multivariable Cox proportional hazard model was created based on predetermined characteristics, including age, sex, and body mass index. A non-parametric mean cumulative function (MCF) was calculated to provide a comprehensive overview when considering multiple recurrent events. These events included: late (>30 days) readmission for RVF, overall readmission, and infection(s) during follow-up, and were presented in a plot [10]. The variance was estimated using the Lawless and Nadeau method [11]. Statistical analyses were performed in R (Version 4.1.2; https://cloud.r-project.org/doc/manuals/r-release/NEWS.html).

## 3. Results

### 3.1 Patient Population

In total, 80 patients were included and encompassed a median follow-up time of 28 months [Interquartile Range (IQR): 18–42] (Table 1). The median age was 57 [IQR: 52–62] years with 21.2% being women. The most frequent aetiology of end-stage heart failure was ischemic heart disease (48%). Patients were mainly in INTERMACS (Interagency Registry for Mechanically Assisted Circulatory Support) profiles 3 and 4 before implantation (26% and 34%). The cardiac rhythm in 53% of the patients was sinus rhythm and 83% of the patients had an implantable cardioverter-defibrillator (ICD) in place. Bridge to transplant was the most prevalent strategy in 60% of cases and 34% of the cases receiving long-term support (destination therapy). Median cardiopulmonary bypass (CPB) time was 95 minutes [IQR: 81–115] and time in the operating room for implantation was 330 minutes [IQR: 280–403]. The median length of intensive care unit (ICU) admission was 8 days [IQR: 5–17] and hospital admission duration was 30 days [IQR: 23–45].

**Table 1.** **Baseline and procedural characteristics**.

		Overall (n = 80)	No PEx (HI <2.0)	PEx (HI 2.0–3.2)	*p* value
			N = 28	N = 52	
Demographics					
	Age in years	57.0 [52.0, 62.0]	59.5 [56.5, 62.0]	56.0 [50.5, 62.5]	0.115
	Men	63 (78.8)	22 (78.6)	41 (78.8)	1.000
	Body mass index	22.9 [20.5, 25.2]	24.8 [22.9, 25.8]	21.7 [18.9, 23.9]	0.001
	Body surface area	2.0 [1.8, 2.1]	2.1 [2.0, 2.2]	2.0 [1.8, 2.1]	0.008
Primary diagnosis					
	Ischemic heart disease	38 (47.5)	19 (67.9)	19 (36.5)	0.019
	Non-ischemic heart disease	42 (52.5)	9 (32.1)	33 (63.5)	
INTERMACS patient profile					
	1	17 (21.2)	3 (10.7)	14 (26.9)	0.096
	2	15 (18.8)	3 (10.7)	12 (23.1)	
	3	21 (26.2)	9 (32.1)	12 (23.1)	
	≥4	27 (33.8)	13 (46.4)	14 (26.9)	
Comorbidities					
	Diabetes	20 (25.0)	10 (35.7)	10 (19.2)	0.176
	ICD therapy	66 (82.5)	24 (85.7)	42 (80.8)	0.805
	Neurological event	5 (6.3)	2 (7.4)	3 (5.8)	1.000
	Smoking	45 (57.0)	14 (51.9)	31 (59.6)	0.673
	COPD	4 (5.0)	1 (3.6)	3 (5.8)	1.000
	Previous cardiac surgery	1 (1.3)	0 (0.0)	1 (1.9)	1.000
Preoperative status					
	Intra-aortic balloon pump	21 (26.2)	4 (14.3)	17 (32.7)	0.129
	Extracorporeal membrane oxygenation	8 (10.0)	1 (3.6)	7 (13.5)	0.310
ECG rhythm					
	Sinus	41 (52.6)	9 (32.1)	32 (64.0)	0.021
	Atrial fibrillation	12 (15.4)	7 (25.0)	5 (10.0)	
	Paced	25 (32.1)	12 (42.9)	13 (26.0)	
	Intravenous inotropes	55 (68.8)	17 (60.7)	38 (73.1)	0.376
Preoperative right ventricular function					
	Stage 1	18	5 (17.9)	13 (25.0)	0.570
	Stage 2	54	21 (75.0)	33 (63.5)	
	Stages 3–4	8	2 (7.1)	6 (11.5)	
Procedural characteristics					
Device strategy					
	Bridge to transplant	48 (60.0)	17 (60.7)	31 (59.6)	0.971
	Destination therapy	27 (33.8)	9 (32.1)	18 (34.6)	
	Other	5 (6.2)	2 (7.2)	3 (5.8)	
Cardiopulmonary bypass time (min)		95.0 [81.0, 115.0]	102.0 [85.5, 113.5]	90.0 [78.0, 115.3]	0.162
Time in operating room for implantation (min)		329.5 [279.8, 402.8]	328.5 [290.0, 402.8]	329.5 [266.5, 402.0]	0.555
ICU stay (days)		8.0 [5.0, 17.0]	7.5 [4.8, 15.5]	8.0 [5.0, 18.5]	0.528
Hospital admission duration (days)		30.0 [23.0, 44.5]	30.0 [23.0, 42.3]	30.0 [23.0, 49.3]	0.600
Length of follow-up (months)		28.3 [18.4, 41.8]	27.6 [15.3, 38.9]	28.3 [19.5, 43.1]	0.420

Continuous variables are described as median [interquartile range (IQR)] and categorical variables as count (percentage). PEx, pectus excavatum; HI, Haller index; INTERMACS, Interagency Registry for Mechanically Assisted Circulatory Support; ICD, implantable cardioverter defibrillator; COPD, chronic obstructive pulmonary disease; ECG, electrocardiogram; ICU, intensive care unit.

### 3.2 Baseline Characteristics

Overall, 52 patients (65%) presented with pectus excavatum (HI 2.0–3.2) whilst 28 patients (35%) had a normal chest-wall with HI <2.0. At baseline, patients with a normal chest-wall had a higher body mass index (*p * = 0.001) and body surface area (*p * = 0.008). The most frequent primary diagnosis was non-ischemic heart disease in patients with pectus excavatum, while patients with a normal chest-wall mainly had ischemic heart disease (*p* = 0.019). Patients with pectus excavatum were more frequently in sinus rhythm whereas a paced rhythm was observed more often in patients with a normal chest-wall (*p * = 0.021) Preoperative right ventricular function did not differ between both groups (*p* = 0.570; Table 1).

### 3.3 Outcomes

**Fig. 2.**
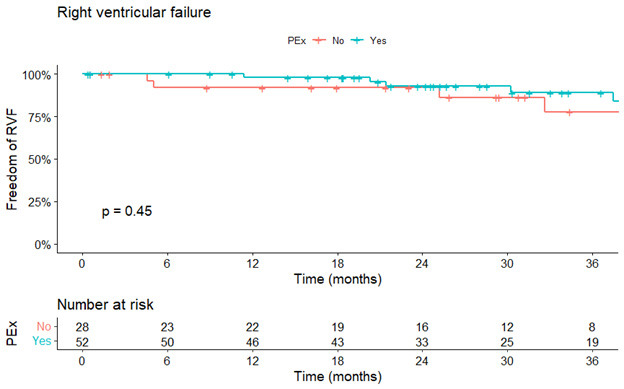
**Kaplan-Meier estimates of right ventricular failure stratified by normal chest-wall (red-line) and pectus excavatum (blue-line)**. PEx, pectus excavatum; RVF, right ventricular failure.

**Fig. 3.**
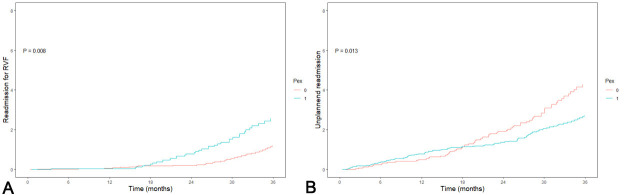
**Mean cumulative function (MCF)**. (A) MCF of right ventricular failure in two groups, with a normal chest (red-line) or a pectus excavatum (blue-line). The Y-axis presents the number of recurrent right ventricular failure and the X-axis represents the time in months. (B) MCF of hospital readmission in two groups, with a normal chest (red-line) or a pectus excavatum (blue-line). The Y-axis presents the number of hospital readmissions and the X-axis represents the time in months. PEx, pectus excavatum; RVF, right ventricular failure; MCF, mean cumulative function.

**Fig. 4.**
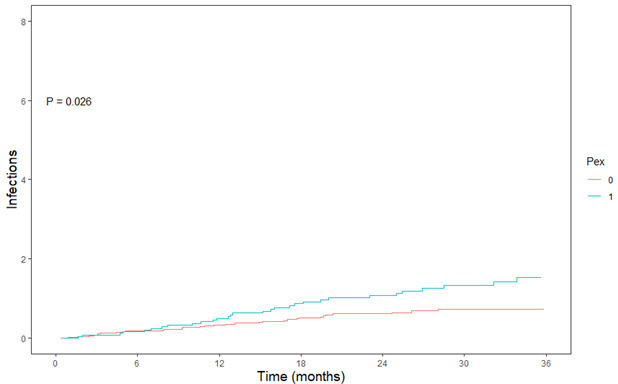
**Mean cumulative function (MCF) of infection in two groups, with a normal chest (red-line) and a pectus excavatum (blue-line)**. The Y-axis presents the number of infections and the X-axis represents the time in months. PEx, pectus excavatum.

**Fig. 5.**
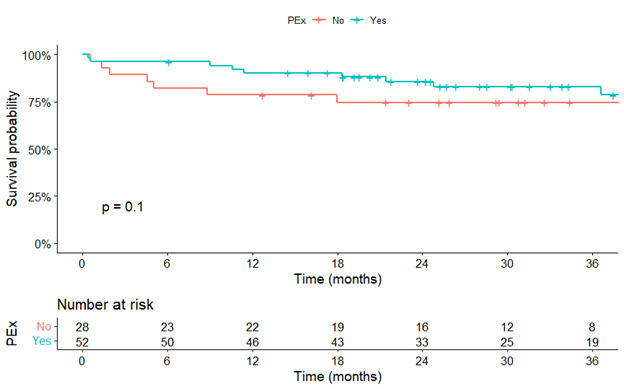
**Kaplan-Meier estimates of survival stratified by normal chest-wall (red-line) and pectus excavatum (blue-line)**. PEx, pectus excavatum.

Early (≤30 days) outcomes including RVF, neurological dysfunction, bleeding, and AKI did not differ between patients with or without pectus excavatum (Table 2). Occurrence of late (>30 days) RVF did not differ between both patient groups with an HR of 0.69 (95% CI: 0.22–2.18, *p * = 0.530) and a log-rank test of *p* = 0.450 (Fig. 2). The primary outcome regarding the occurrence of RVF was not met, however, an exploratory analysis of the number of readmissions for RVF was performed. Readmission(s) for RVF is a potential recurring event and therefore a mean cumulative function was calculated considering multiple events per patient during follow-up. Patients with pectus excavatum had significantly more readmissions for late RVF during follow-up (*p * = 0.008; Fig. 3A). The onset of readmission for late right ventricular failure started around 18 months of follow-up and increases thereafter in the overall study population (Fig. 3A). In total there were 198 unplanned readmissions, with 90 (45%) readmissions for right heart failure, 73 (37%) for infection, 25 (13%) for bleeding complications, 8 (4%) for neurological dysfunction and 2 (1%) other. All-cause readmissions occurred more in patients with a normal chest-wall, with 1.29 versus 0.65 readmission(s) per person year during follow-up (*p* = 0.013; Fig. 3B). Readmission for recurrent infection occurred significantly more in patients with pectus excavatum, with 0.49 versus 0.30 readmission(s) for infection per person per year during follow-up (*p* = 0.026; Fig. 4). There was no significant survival difference between the two groups of patients (HR: 0.47, 95% CI: 0.19–1.19, *p* = 0.113; log-rank test of *p* = 0.100 (Fig. 5)). Bleeding, neurological dysfunction, and chronic kidney disease did not differ in the two groups, respectively an HR of 1.29 (95% CI: 0.45–3.66, *p* = 0.634), an HR of 2.30 (95% CI: 0.27–19.68, *p* = 0.448) and an HR of 0.77 (95% CI: 0.43–1.38, *p* = 0.382; Table 2). Multivariable analysis showed a significant difference between chronic kidney disease when adjusting for age. All other late postoperative outcomes, when adjusting for age, gender, and body mass index, did not show significant differences (**Supplementary Table 1**).

**Table 2.** **Univariable logistic regression and Cox regression of early (<30 days) and late (>30 days) clinical outcomes of patients compared by chest-wall, with or without pectus excavatum**.

		No PEx	PEx	${\mathrm{OR}}^{1}$ / ${\mathrm{HR}}^{2}$	95% CI	*p*-value
		N = 28	N = 52			
Early (<30 days)						
	Early right ventricular failure 1	12	17	0.65	0.25–1.68	0.370
	Early neurological dysfunction 1	2	0	-	-	-
	Early bleeding 1	7	19	1.73	0.64–5.06	0.290
	Acute kidney injury 1	14	18	0.53	0.21–1.35	0.180
Late (>30 days)						
	Late right ventricular failure 2	5	7	0.69	0.22–2.18	0.530
	Late Neurological dysfunction 2	1	5	2.30	0.27–19.68	0.448
	Late bleeding 2	5	12	1.29	0.45–3.66	0.634
	Chronic Kidney Disease (eGFR <${\mathrm{60)}}^{2}$	18	31	0.77	0.43–1.38	0.382

PEx, pectus excavatum; ${\mathrm{OR}}^{1}$, ${\mathrm{odds ratio}}^{1}$ (logistic regression); ${\mathrm{HR}}^{2}$, ${\mathrm{hazard ratio}}^{2}$ (Cox regression); CI, confidence interval; eGFR, estimated glomerular filtration rate (milliliters per minute).

## 4. Discussion

In this study, we investigated the role of pectus excavatum on adverse outcomes including RVF in patients undergoing LVAD support. Overall survival and early or late RVF did not differ in the two groups. Although the unplanned readmission rate was higher in patients with normal chest wall, an increased readmission rate due to RVF and recurrent infection was observed in patients with pectus excavatum after 18-month follow-up.

### 4.1 Pectus Excavatum

Pectus excavatum accounts for approximately 90% of all chest-wall abnormalities and has an incidence of 1 in 400 to 1 in 1000, with men 3 to 5 times more affected than women [16]. The prevalence of pectus excavatum in our study was 65%. This rather high prevalence may be due to the sensitivity of the HI whereas our patients only had a normal chest-wall (HI <2.0) or a mild pectus excavatum (HI 2.0–3.2). For example, surgical intervention is considered in patients with severe pectus excavatum (with a HI of 3.25 or more) [17]. Risk factors for pectus excavatum include Marfan syndrome, Ehlers-Danlos syndrome, and a familial risk [18, 19]. Risk factors for pectus excavatum are similar to those for dilated cardiomyopathy, which include a tall and slender build [20]. This may explain the lower body mass index and body surface area with more presence of dilated cardiomyopathy in patients with pectus excavatum. Patients with previous (congenital) cardiac surgery may have an increased risk of developing pectus excavatum [21, 22]. Our study however included only one such patient, making subgroup analysis not feasible. Hence, we decided to use both pre-operative and postoperative scans based on availability.

### 4.2 Physical Exam and Imaging

Physical examination is an important part of the initial diagnosis of a chest-wall abnormality [23]. When LVAD therapy is being considered, a full thorough physical examination should be performed to provide a clear overview of the patient’s physical state and body shape, even if the patient is a tertiary referral. When a physician suspects a chest-wall abnormality may present, or even if the patient has one or more of the aforementioned risk factors for a chest-wall abnormality, advanced imaging should be considered. A CT scan of the chest should be performed and the Haller index calculated [24]. This allows the cardiac surgeon to plan the operation and take any chest well abnormalities into account when placing the LVAD. For example, they can angle the outflow graft more toward the right and thereby minimizing the risk of compression of the right ventricle and thereby preventing the development of RVF.

### 4.3 Right Ventricular Failure

Our study is consistent with earlier research findings that also reported a high incidence of RVF in their study populations in the early phase (≤30 days) after LVAD implantation [7, 25, 26]. Early RVF is often transient in nature and not associated with long-term reduced survival [25, 27]. On the other hand, late-onset RVF (>30 days) tends to be more persistent and is associated with a greater risk of mortality in patients supported by LVADs. As a result, it often requires multiple hospital readmissions [5, 6, 28]. Late RVF did not show a significant difference between patients with and without pectus excavatum. This result may be due in part to the population considered in our study, which only consisted of patients with a normal chest-wall or a mild pectus excavatum (defined as a low HI). Severe pectus excavatum may compress the right heart and various components of the LVAD, potentially impacting LVAD function. This compression can occur specifically on the outflow graft, leading to constriction of the right ventricle and compromised inflow. This compression would likely contribute to RVF and right-sided congestion [29, 30].

Readmission for late RVF occurred significantly more in the patients with a pectus excavatum and increased during follow-up in both groups. One notable finding was the specific time of onset of hospital readmission for late RVF in the overall cohort, at 18 months of support. The time of onset of late RVF has been studied earlier and varying widely, from 30 days to 1798 days after LVAD implantation [28]. The underlying causes of late RVF during LVAD support are diverse and often multifactorial [28]. Our study identifies a potential new risk factor for late RVF for LVAD-supported patients, as patients with a pectus excavatum had more unplanned readmissions, suggesting an increased severity of RVF. This finding emphasizes the importance of properly assessing the chest wall in LVAD candidates and possibly adjusting the course of the LVAD outflow graft over the right ventricle. Furthermore, periodic assessment of right ventricular function may decrease the incidence of unplanned hospital readmissions in this population.

### 4.4 Adverse Events

This study demonstrated a higher occurrence of infections in the pectus excavatum group during follow-up, which was unexpected given the lack of literature regarding a higher infection risk in patients with a mild pectus excavatum compared to those with a normal chest wall. It is possible that other underlying conditions, not related to the chest wall, may contribute to these differences. The most common cause of unplanned readmissions for LVAD-supported patients are infections, particularly those related to the driveline [31]. Despite the higher occurrence of infections in patients with pectus excavatum, the normal chest-wall group had a higher number of unplanned readmissions. A possible explanation for this finding could be the significantly higher prevalence of ischemic heart disease as the primary diagnosis in the normal chest-wall group, indicating a higher prevalence of systemic arterial vascular disease and increased frailty. However, this hypothesis has previously been investigated, and no differences were found in outcomes when comparing ischemic heart failure to non-ischemic heart failure in previous studies [32]. Further research is needed to identify other factors that may contribute to differences in overall unplanned hospital readmission rates.

## 5. Limitations

The results of this retrospective single-center study should be interpreted in the context of several limitations. The patients in the study cohort had a normal chest-wall (HI <2.0) or a mild pectus excavatum (HI 2.0–3.2), with no patients with a moderate or severe pectus excavatum. The findings should be interpreted with caution since the small sample size limits the power of the study and represent an increased risk of bias. Despite this issue, this study presents novel findings regarding the impact of chest-wall morphology during LVAD support. Although the HI is, in our opinion, generally considered the most reliable metric for assessing the severity of pectus excavatum, the use of different indices in the current literature may limit the generalizability of the outcomes of this study. Ideally, patients undergo a pre- and postoperative CT scan of the chest to evaluate the chest-wall and determine the influence of the operation itself. However, the literature suggests there is little difference in the occurrence of a pectus excavatum following cardiac surgery. Finally, this study did not specifically analyze hemodynamic influences, such as changes in echocardiographic function measurements over time, in patients with pectus excavatum compared to those without chest wall abnormalities. Despite this limitation, this study examined both early and late RVF outcomes, providing a novel and valuable insight for clinical practice.

## 6. Conclusions

This study found no significant association between pectus excavatum and early or late RVF after LVAD implantation. However, readmission for late RVF occurred significantly more often in patients with a pectus excavatum with a specific time of onset of 18 months or later post-LVAD implantation and increased hereafter. This suggests the importance of evaluating right ventricular function during follow-up and highlights the need for further research into the underlying mechanisms of RVF.

## Data Availability

All relevant anonymized data is available from the authors on request.

## Supplementary Material

Supplementary material associated with this article can be found, in the online version, at https://doi.org/10.31083/j.rcm2411313.
