# Supplementary material for: Pectus Excavatum and Risk of Right Ventricular Failure in Left Ventricular Assist Device Patients
Source: Rev Cardiovasc Med. 2023 Nov 9;24(11):313. doi: 10.31083/j.rcm2411313 (PMC11272874; doi:10.31083/j.rcm2411313)
Supplement: Supplementary file 1 [file 2153-8174-24-11-313-s1.docx]

**SUPLLEMENTARY MATERIAL**

**Table 1.** Multivariable analysis cox proportional hazards model of right ventricular failure, mortality, neurological dysfunction, bleeding and chronic kidney disease (eGFR <60). eGFR: estimated Glomerular Filtration Rate in milliliters per minute).

| **Variable** | **Hazard Rate (HR)** | **CI, lower 95%** | **CI, upper 95%** | ***P* value** |
| --- | --- | --- | --- | --- |
| **Right ventricular failure** | | | | |
| Pectus excavatum | 0.57 | 0.15 | 2.15 | 0.404 |
| Age in years | 1.00 | 0.95 | 1.05 | 0.992 |
| Gender, male | 0.85 | 0.22 | 3.27 | 0.817 |
| Body Mass Index | 0.94 | 0.80 | 1.11 | 0.445 |
| **Mortality** | | | | |
| Pectus excavatum | 0.37 | 0.12 | 1.18 | 0.092 |
| Age in years | 1.05 | 0.98 | 1.12 | 0.153 |
| Gender, male | 0.45 | 0.16 | 1.26 | 0.130 |
| Body Mass Index | 0.94 | 0.82 | 1.09 | 0.426 |
| **Neurological dysfunction** | | | | |
| Pectus excavatum | 2.21 | 0.23 | 20.82 | 0.488 |
| Age in years | 0.99 | 0.93 | 1.06 | 0.781 |
| Gender, male | 0.55 | 0.10 | 3.04 | 0.492 |
| Body Mass Index | 1.00 | 0.81 | 1.24 | 0.985 |
| **Bleeding** | | | | |
| Pectus excavatum | 1.56 | 0.49 | 5.01 | 0.453 |
| Age in years | 1.03 | 0.98 | 1.09 | 0.297 |
| Gender, male | 0.62 | 0.21 | 1.80 | 0.380 |
| Body Mass Index | 1.02 | 0.89 | 1.17 | 0.779 |
| **Chronic Kidney Disease** | | | | |
| Pectus excavatum | 0.97 | 0.50 | 1.89 | 0.943 |
| Age in years | 1.04 | 1.01 | 1.08 | 0.006 |
| Gender, male | 0.82 | 1.22 | 1.68 | 0.586 |
| Body Mass Index | 1.00 | 1.00 | 1.09 | 0.973 |
